# Supplementary material for: Nano TiO2 and Molybdenum/Tungsten Iodide Octahedral Clusters: Synergism in UV/Visible-Light Driven Degradation of Organic Pollutants
Source: Nanomaterials (Basel). 2022 Dec 1;12(23):4282. doi: 10.3390/nano12234282 (PMC9736415; doi:10.3390/nano12234282)
Supplement: Supplementary file 1 [file nanomaterials-12-04282-s001.zip › nanomaterials-2074070-supplementary.pdf]

Supporting Information

Nano TiO_2 and Molybdenum/Tungsten Iodide Octahedral Clusters: Synergism in UV/Visible-Light Driven Degradation of Organic Pollutants

Margarita V. Marchuk [1], Igor P. Asanov [1], Maxim A. Panafidin [2], Yuri A. Vorotnikov [1,*] and Michael A. Shestopalov [1]

[1] Nikolaev Institute of Inorganic Chemistry SB RAS, 3 Academician Lavrentiev Avenue, 630090 Novosibirsk, Russia
[2] Boreskov Institute of Catalysis SB RAS, 5 Academician Lavrentiev Avenue, 630090 Novosibirsk, Russia
* Correspondence: vorotnikov@niic.nsc.ru.

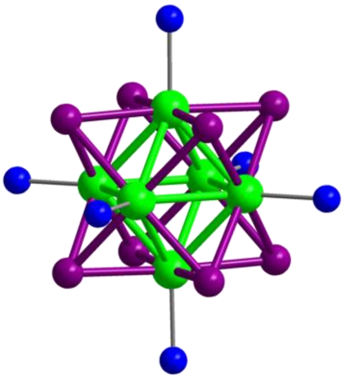

Figure S1. Representative structure of $[\{M_6I_8\}L_6]^n$ (M = Mo or W; green octahedron is M_6, violet spheres are inner iodine ligands, blue speres are apical ligands (L) of any nature, n – charge) units.

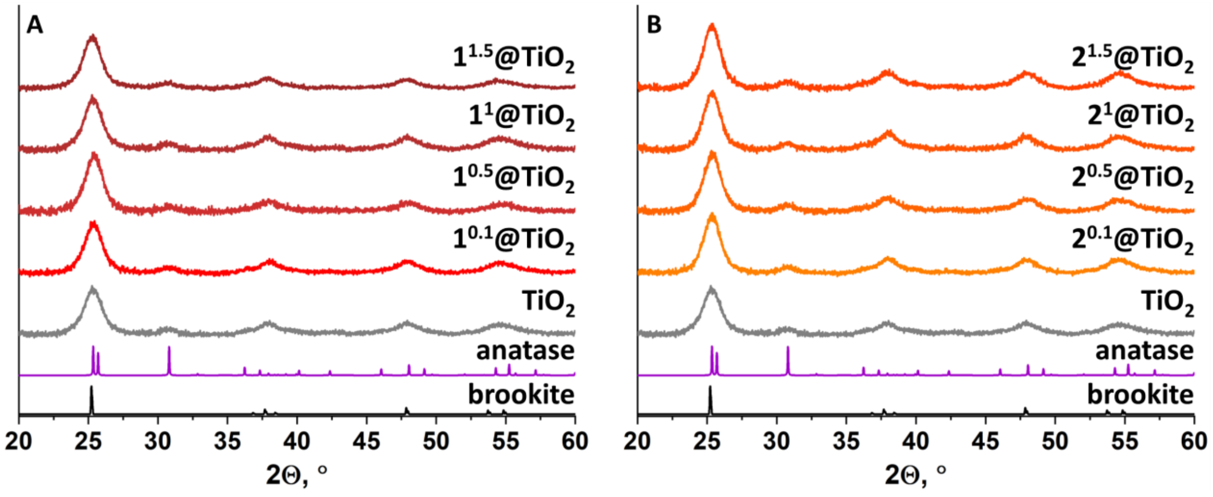

Figure S2. XRD patterns of n^x@TiO_2, n = 1 (A) or 2 (B) in comparison with pure TiO_2 and calculated anatase and brookite diffractograms.

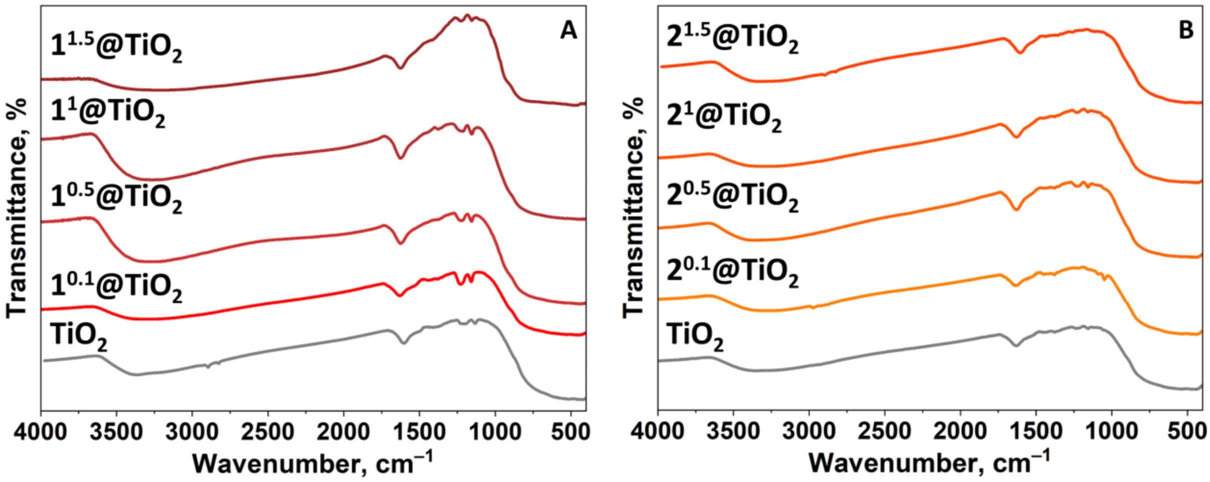

Figure S3. FTIR spectra of pure TiO_2 and n^x@TiO_2, n = 1 (A) or 2 (B).

Table S1. Determination of the amount of {M_6I_8} units anchored on TiO_2 using ICP-AES.

Material	n	M content, w%	{M_6I_8} content, mmole({M_6I_8}) g(TiO_2)$^{-1}$
**1**^{n}@TiO_2	0.1	0.16 % wt.	0.0031
	0.5	0.27 % wt.	0.0046
	1	0.26 % wt.	0.0044
	1.5	0.29 % wt.	0.0050
**2**^{n}@TiO_2	0.1	1.3 % wt.	0.012
	0.5	3.2 % wt.	0.03
	1	5.2 % wt.	0.05
	1.5	8.0 % wt.	0.079

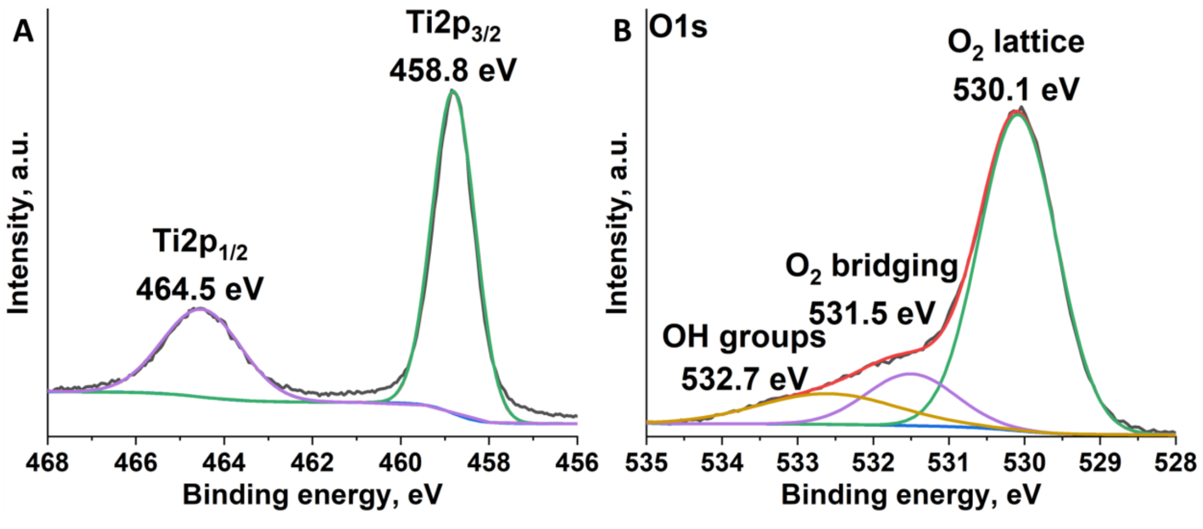

Figure S4. High-resolution XPS spectra of Ti2p (A) and O1s (B) core levels in $2^{0.1}$@TiO_2.

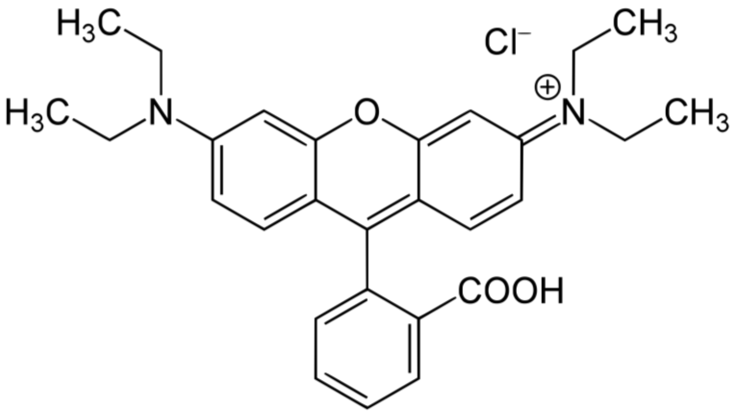

Figure S5. Structure of RhB molecule.

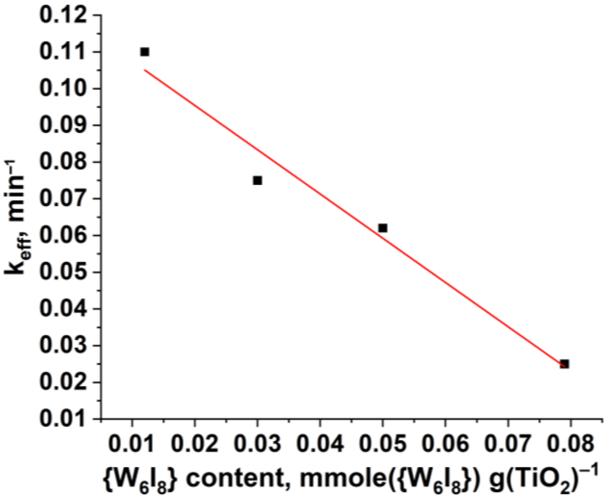

Figure S6. The dependency of k_{eff} of $2^x@TiO_2$ on real content of $\{W_6I_8\}$ determined using ICP-AES.

Table S2. Effective rate constants (k_{eff}) and R^2 values of RhB decomposition by $n^x@TiO_2$ in the presence of different scavengers.

**Scavenger**	**$1^x@TiO_2$**			**$2^x@TiO_2$**		
	**k_{eff}, min^{-1}**	**R^2**	**RA#, %**	**k_{eff}, min^{-1}**	**R^2**	**RA#, %**
No scavengers	0.099	0.99019	100	0.11	0.97244	100
^{i}PrOH	0.088	0.99728	89	0.096	0.98827	87
$AgNO_3$	0.019	0.99198	19	0.036	0.97546	33
$Na_2C_2O_4$	0.028	0.9793	28	0.047	0.99003	43
Ar	0.015	0.97798	15	0.01	0.97153	9

#Relative activity (RA) was calculated according to the following formula: $RA = \frac{k_{eff(scav)}}{k_{eff(NS)}} \times 100\%$, where k_{eff}(scav) is effective rate constant in the presence of a certain scavenger, k_{eff}(NS) is effective rate constant in scavenger free experiment.
